# Ultrasonographic study of female perineal body and its supportive function on pelvic floor

**DOI:** 10.3389/fmed.2023.1176360

**Published:** 2023-07-26

**Authors:** Minzhi Zhou, Wen Shui, Wenkun Bai, Xing Wu, Tao Ying

**Affiliations:** Department of Ultrasound in Medicine, Shanghai Institute of Ultrasound in Medicine, Shanghai Sixth People’s Hospital Affiliated to Shanghai Jiao Tong University School of Medicine, Shanghai, China

**Keywords:** high-frequency two-dimensional ultrasound, female perineal body, shear wave elastography, Young’s modulus, pelvic floor dysfunction

## Abstract

**Objectives:**

The study aimed to observe, measure the size and elastic value of perineal body (PB) and assess its association with levator hiatus.

**Methods:**

Datasets were acquired in 45 nulliparous, 66 POP women and 70 postpartum women using ultrasound. The PB was measured in depth, height, and Young’s modulus. The datasets were compared to assess whether there are some differences in the morphology, dimension and elastography modulus of PB among women. Pearson correlation analysis was used to evaluate the association between the morphology measurements (ΔValsalva-rest[v-r]), tissue mechanical properties (ΔValsalva-rest[v-r]) of the PB and levator hiatus area (ΔValsalva-rest[v-r]) to preliminarily explore whether PB can influence levator hiatus.

**Results:**

Four representative manifestations of PB were presented in our study. Nulliparous women had smaller diameters and bigger Young’s modulus while postpartum women had bigger diameters and smaller Young’s modulus. POP and postpartum women had bigger levator hiatal distensibility and PB extensibility. There was no statistical association between PB measurements and levator hiatal area.

**Conclusion:**

It is feasible to observe the morphology of PB and assess the dimension and elastography modulus by high-frequency ultrasound. The manifestations and measurements of PB are influenced by parity and long-term increased abdominal pressure. Our study preliminarily shows that PB has little effect on levator hiatus area.

## 1. Introduction

Pelvic organ prolapse (POP) is a common condition among women, especially in aged women over 50 years or young postpartum women with prolonged second birth labor in vaginal delivery. The pathophysiology of POP is still unclear, and it may be related to risk factors such as pregnancy, vaginal parity, inherent weakness, and growing age (1–4). Pelvic floor is a complex structure composed of organs, bones, muscles, fascia, ligaments, and other supportive tissues (5). The perineal body (PB), the third supportive level of the pelvic floor, is a complex fibromuscular structure adherent anteriorly to the vaginal muscularis and connected to the posterior border of the perineal membrane. It plays an important role in maintenance of the integrity of pelvic floor and the normal morphology of levator hiatus (6). Vaginal delivery-related trauma to the PB has been shown to occur in 10%–30% of women delivering vaginally (7–9). Once the PB is injured, it will undergo morphological and mechanical changes, and thus its supportive effect on pelvic floor will be decreased, leading to the descent of pelvic organs and the abnormal distensibility of levator hiatus (6).

The transperineal ultrasound with abdominal transducer has been used to scan the PB. In previous studies, the PB was measured on images that contained the entire pelvic floor, so the image quality was poor and the measurement was less repeatable and inaccurate (10). Endovaginal or endoanal ultrasound has emerged as a readily available alternative to demonstrate the PB (11–14), but it demands high technical requirements. Magnetic resonance imaging (MRI) has been extensively used to assess the posterior pelvic compartment, however it is not possible to delineate the PB with clarity for detailed assessment of dimensions due to its small size and anatomical location (15). In former studies, high-frequency two-dimensional (2D) ultrasound has been used to detect muscle avulsion and evaluate biological function of levator ani muscles by elastography technology (16, 17). Shear wave elastography (SWE) technology is an innovative technology used to evaluate tissue mechanical properties during ultrasound examination. The advantage of this imaging modality is that the mechanical impulse is operator independent and the tissue stiffness can be quantitatively measured; therefore, objective tissue stiffness quantification can be achieved. The PB was in a superficial position under perineum membrane, so we raised the hypothesis that high-frequency 2D ultrasound could show a clear image of it. After repeated clinical practice, we found high-frequency ultrasound could demonstrate PB with more details such as fibers and measure the dimension more conveniently when compared with abdominal transducer. In this study, we attempted to demonstrate a clear image and make a quantitative assessment of the dimension and elastography modulus of the PB. The datasets were compared between nulliparous women, postpartum women and POP women to assess whether the morphology, dimension and elastography modulus of the PB varied among women in order to have a deep understanding of the PB. The injured pelvic supportive structures were related to the descent of pelvic organs and the abnormal distensibility of levator hiatus, so in this study we first attempted to find out whether PB was associated with levator hiatus area (HA).

## 2. Materials and methods

### 2.1. Patients

This retrospective study was approved by 2022-KY-142(K) by the Ethics Committee of Shanghai Sixth People’s Hospital Affiliated to Shanghai Jiao Tong University School of Medicine. Written informed consent was obtained from every woman who underwent ultrasonography. A total of 192 women who went to the urological and gynecological clinic of the hospital for consultation of irregular menstruation, vaginitis, infertility, pelvic floor discomfort, and postpartum 42 days follow-up were included. Exclusion criteria were: (1) women with prior pelvic surgery; (2) women who did not want to have a pelvic examination; and (3) women who were unable to perform an adequate muscle contraction or Valsalva maneuver after training. All of the women had a clinical POP-Q assessment and ultrasound scan. We collected the following anthropometric data and socio-demographic data: age, body mass index (BMI), and times of parity.

### 2.2. Ultrasound examination

**Figure 1:**
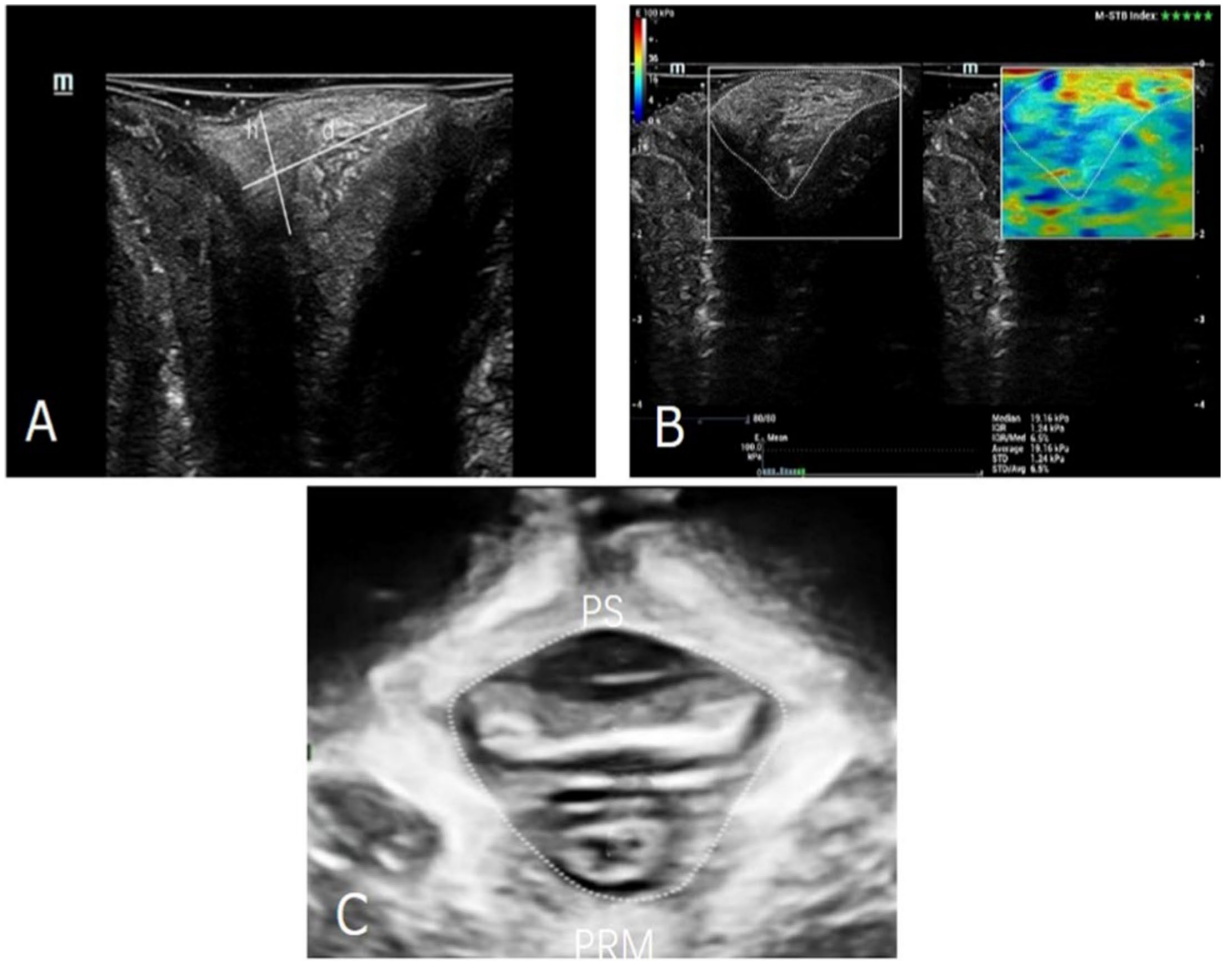
**(A)**. The diameters of the PB were outlined. h, the cranio-caudal diameter; d, was the antero-posterior diameter. **(B)** The Young’s modulus of the PB was measured by SWE technology. **(C)** The area of levator hiatus was outlined. PS, pubis symphysis; PRM, puborectalis muscle.

We used a Mindray expert system (Resona 7S Medical System, China) with 10.0 MHz linear transducer equipped with SWE software. Ultrasound examination was performed gently by an experienced doctor, who was blinded to all clinical information. Imaging was performed with the probe placed on the PB without any pressure on the tissue, as excessive pressure applied by the probe could potentially interfere with measurements. The PB lay interposed subcutaneously between the vagina and the anal canal. The height (h) of the PB was the cranio-caudal diameter and the depth (d) was the antero-posterior diameter 13 (Figure 1A). The border of the PB was outlined by hand, and the mean Young’s modulus (Y) was obtained within the border on SWE mode (Figure 1B). All measurements were repeated three times, and the mean value was calculated and saved. All ultrasound datasets were obtained for each woman under three different states of abdominal pressure, including at rest, on maximal muscle contraction and on Valsalva. The difference value of height, depth and Young’s modulus at rest and on Valsalva (Δrv-h, Δrv-d and Δrv-Y) were also analyzed, which were used to evaluate the degree of PB extensibility.

Then three-dimensional transperineal ultrasound was performed using a Mindray expert system (Resona 7S Medical System, China) with RAB4-8-D volume probe. Relevant levator hiatus area was measured at the minimal axial plane identified between the hyperechoic posterior aspect of the pubic symphysis and the hyperechoic anterior border of the puborectalis muscle (Figure 1C). The Δrv-HA (ΔValsalva-rest[v-r]) values was also analyzed, which were used to evaluate the degree of the levator hiatus distension.

### 2.3. Statistical analysis

Statistical analysis was undertaken using SPSS 22.0 software for Windows (SPSS Chicago, IL, United States). The measurements were expressed as mean ± standard deviation. ANOVA analysis was performed to evaluate the differences of the height (h), depth (d) and Young’s modulus (Y) among different groups. Pearson correlation analysis was used to evaluate the association between the morphology measurements(Δrv-h and Δrv-d),tissue mechanical properties (Δrv-Y) of the PB and levator hiatus area (Δrv-HA). The value of *p* < 0.05 was considered of statistical significance.

## 3. Results

### 3.1. Demographics

Of the all 192 datasets, 11 were excluded from analysis for poor image quality or levator ani muscles avulsion, leaving valid data for 181 women, including 45 healthy non-pregnant nulliparous women, 66 POP women and 70 postpartum women. The 181 women’s clinical information was shown in Table 1. Nulliparous women had an average age of 30.2 years and BMI 22.6. POP women had an average age of 51.2 years and BMI 23.8. Postpartum women had an average age of 31 years and BMI 23.4. A total of 46 (69%) POP women and 36 (51%) postpartum women had spontaneous vaginal deliveries. A total of 31 (47%) POP women and 19 (27%) postpartum women had two or more deliveries. In POP women, 52 had a cystocele, 5 had a uterine prolapse, 6 had a cystocele combined with uterine prolapse, and 3 had a rectocele. Women who recently delivered within 42 days were included into the postpartum group no matter they had prolapse or not.

**Table 1:** Demographic data of women in different groups.

Variable	Nulliparous	POP	Postpartum
Age, *y*	30.20 ± 6.96	51.17 ± 16.32	30.97 ± 4.38
BMI, kg/m	22.60 ± 3.54	23.76 ± 3.36	23.42 ± 3.45
Parity, *n*	0	1.64 ± 0.91	1.35 ± 0.54
Pelvic organ prolapse grade, *n*			
0	45	0	51
I	0	3	4
II	0	32	13
III	0	25	2
IV	0	6	0

### 3.2. The manifestations of PB among different groups

**Figure 2:**
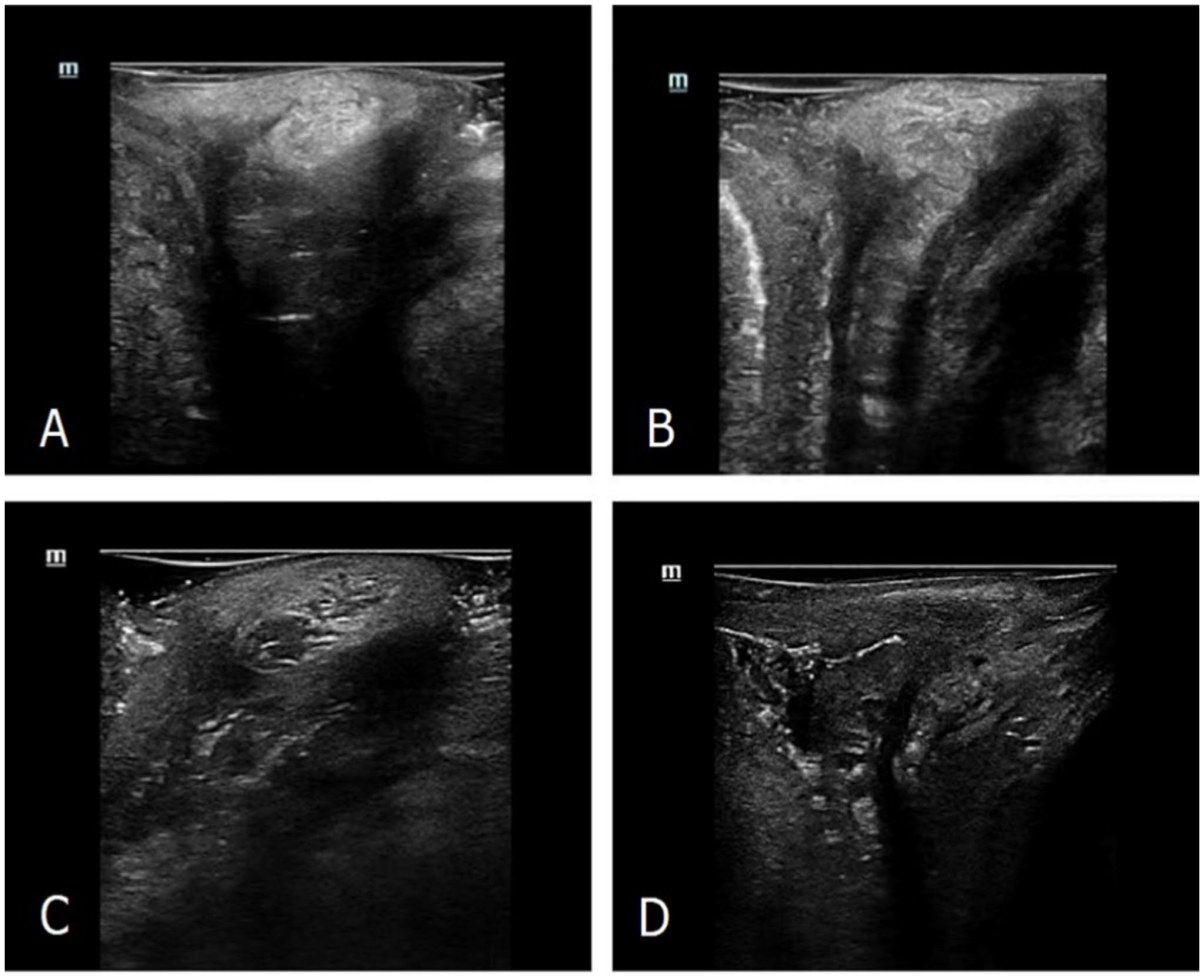
Ultrasonographic manifestations of the PB on high-frequency ultrasound. **(A)** The PB morphology of representative nulliparous women detected by ultrasound. **(B)** The PB morphology of representative parous and POP women detected by ultrasound. **(C)** The PB morphology of representative postpartum women detected by ultrasound. **(D)** The ultrasonic image of very few postpartum women with severe perineal laceration.

Four representative manifestations of the PB were obtained. The image of the PB in most nulliparous women was visualized as a relatively even hyperechoic structure with triangular shape in the mid-sagittal plane (Figure 2A). Most POP women had PB images with more blurred borders and rougher echoes than those of nulliparous women (Figure 2B). Ultrasound images of the PB in a small part of POP women were similar to those of nulliparous women. Of the most postpartum women, the PB was visualized as an inhomogeneous echo structure with little hypoechoic area on an ultrasound image (Figure 2C). The hyperechoic structure was not obtained in postpartum women with severe perineal laceration (Figure 2D).

### 3.3. The depth and height parameters of PB among different groups

Tables 2, 3 show the comparison of PB parameters among groups under different conditions, including at rest, on maximal muscle contraction and on Valsalva. The depth and height diameters were found to be significantly increased on contraction (*p* < 0.05). The depth and height diameters of PB in postpartum group were bigger than other groups (*p* < 0.05). The differences were of statistical significance (*p* < 0.05).

**Table 2:** Comparison of the PB depth parameters among groups.

Group	r-d (mm)	c-d (mm)	v-d (mm)
Nulliparous	14.24 ± 2.85	16.70 ± 3.07	14.67 ± 2.62
POP	14.76 ± 2.65	16.86 ± 3.19	15.74 ± 2.68
Postpartum	16.65 ± 2.66	18.95 ± 2.91	17.44 ± 3.28
*p* value (Nulliparous vs. POP)	0.337	0.795	0.079
*p* value (Nulliparous vs. Postpartum)	<0.005	<0.005	<0.005
*p* value (POP vs. Postpartum)	<0.005	<0.005	<0.005

r-d, depth diameter at rest; c-d, depth diameter on contraction; v-d, depth diameter on Valsalva.

**Table 3:** Comparison of the PB height parameters among groups.

Group	r-h (mm)	c-h (mm)	v-h (mm)
Nulliparous	7.78 ± 1.44	8.32 ± 1.51	7.55 ± 1.41
POP	7.41 ± 1.19	8.08 ± 1.31	7.17 ± 1.06
Postpartum	8.37 ± 1.24	8.97 ± 1.22	8.27 ± 1.31
*p* value (Nulliparous vs. POP)	0.123	0.352	0.115
*p* value (Nulliparous vs. Postpartum)	<0.05	<0.05	<0.005
*p* value (POP vs. Postpartum)	<0.005	<0.005	<0.005

r-h, height diameter at rest; c-h, height diameter on contraction; v-h, height diameter on Valsalva.

### 3.4. The Young’s modulus of PB among different groups

Table 4 shows the comparison of the PB Young’s modulus among women under different conditions, including at rest, on maximal muscle contraction and on Valsalva. Overall, nulliparous women had bigger Young’s modulus than other groups (*p* < 0.05). In every group, the Young’s modulus were found to be significantly increased on contraction (*p* < 0.05). The differences were of statistical significance (*p* < 0.05).

**Table 4:** Comparison of the PB Young’s modulus (Y) among groups.

Group	r-Y (kPa)	c-Y (kPa)	v-Y (kPa)
Nulliparous	23.80 ± 3.76	34.42 ± 4.71	25.00 ± 3.70
POP	21.24 ± 2.90	28.77 ± 2.55	22.28 ± 2.50
Postpartum	20.98 ± 2.52	28.77 ± 2.44	21.58 ± 2.73
*p* value (Nulliparous vs. POP)	<0.001	<0.001	<0.001
*p* value (Nulliparous vs. Postpartum)	<0.001	<0.001	<0.001
*p* value (POP vs. Postpartum)	0.595	0.991	0.153

r-Y, Young’s modulus at rest; c-Y, Young’s modulus on contraction; v-Y, Young’s modulus on Valsalva.

### 3.5. Correlation between PB measurements and levator hiatus area

To explore whether the change of the PB would affect the dimension of levator hiatus synchronously, we performed Pearson’s analysis to evaulate the relationship between the PB measurements and levator hiatus area. The morphology measurements (Δrv-d and Δrv-h) and tissue mechanical properties (Δrv-Y) of the PB were not associated with the levator hiatus area (Δrv-HA) (*p* > 0.05).

## 4. Discussion

The PB plays a key role in the normal structure and function of female pelvic floor. It is often subjected to trauma during vaginal childbirth and also be influenced by increased abdominal pressure (18, 19). Imaging researches have been helpful in the study of the PB, the transperineal ultrasound with abdominal transducer, endovaginal and endoanal ultrasound have been used to assess the PB in recent years. However, some examination modes have a high demand on the technique of operation. A simple and widely available method of assessing the PB may be of great clinical value.

In this study, we successfully applied high-frequency 2D ultrasound, a technique which is good at displaying superficial tissue of the pelvic floor, in imaging the morphology of the PB, and generalized representative manifestations in three groups. We noted that in most postpartum women, the PB was visualized as an inhomogeneous hyperechoic structure with little hypoechoic area on an ultrasound image, which is an expected result as it reflects the changes in internal structure of the PB after pregnancy and delivery (20). We speculate that the inhomogeneous hyperechoic appearance may be related with loose tissue, unabsorbed hematoma or immature granulation tissue. In very few postpartum women with severe perineal laceration, the region of the PB was visualized as an irregular hypoechogenic zone rather than a hyperechoic structure. This manifestation is similar to the presentation of levator ani muscle avulsion (17). Furthermore, the PB images of most POP women had blurry borders and rough echoes, and we supposed that it might imply the long-term chronic repair of the PB itself after injury. However, further study is needed to provide sufficient evidence of the cause and pathomechanism of this change. It is possible that the condition of PB injury and recovery can be detected through an ultrasound examination, which gives more internal information than clinical inspection and palpation. With high-frequency 2D ultrasound, a simple, non-invasive examination, we acquired clear images that were optimised specifically for the PB. Optimised images enable us to demonstrate the PB with more details and detect some abnormal performance.

Furthermore, obvious differences were found in the dimensions and Young’s modulus of the PB among three groups. Nulliparous women had smaller diameters and bigger Young’s modulus while postpartum women had bigger diameters and smaller Young’s modulus. These findings were expected results as they reflected pregnancy and delivery led to the changes of the morphology and tissue mechanical property of the PB to some extent. In addition, in postpartum group, the results showed no statistically significant difference between women delivered vaginally and those delivered by cesarean section. This finding may imply that the effect of hormone and other internal factors rather than delivery mode is more responsible for changes of the PB, which is consistent with a reported study (21). The PB has certain extensibility, and it will undergo morphological and mechanical changes to resist the increased abdominal pressure. Asfour et al. raised that the PB is an important supportive structure for the pelvic floor and its morphological differentiation may lead to pelvic floor disorders (22). In this study, POP women and postpartum women had bigger extensibility diameters. We speculated that the PB of POP or postpartum women may be a destabilizing factor in itself, putting muscles and fascial collagenous structures under strain, causing greater deformation. Under the combined application of 2D high-frequency ultrasound and SWE technology, it is convenient to display the morphology and elastic module of the PB, showing the internal features of the PB to some extent.

Anatomically, the dimension of levator hiatus is delimited by pelvic supportive structures, thus, its abnormal distensibility can be reasonably considered as negative effects of the defected supportive structures. As the perineal membrane and tendinous arches of the pelvic fasciae tighten, the PB redirects abdominal pressure and disperses gravity to prevent the distensibility of levator hiatus (23). In our study, POP and postpartum group had bigger levator hiatal distensibility and PB extensibility, which is an expected change as it reflects loose structure of the pelvic. In addition, our study showed there was no statistically significant correlation between the morphology measurements (Δrv-d and Δrv-h) and Young’s modulus (Δrv-Y) and levator hiatus area (Δrv-HA). For the whole pelvic floor, the PB is located at the superficial part and has a relatively small share, not forming a V-shaped sling to prevent expansion of levator hiatus like puborectalis muscles. We supposed that the small size and superficial anatomical location of PB may explain the negative result in this study. However, further larger sample size studies are needed to provide sufficient evidence.

Our study had some limitations that could not be ignored, so the results should be interpreted carefully. As the sample size was small, we consider it a preliminary study, so the accuracy and efficiency should be confirmed in further investigations with larger sample size. Furthermore, the measuring method of the PB on high-frequency ultrasound was different from former studies, so the measurements required to be further verified with a standard and general method. In this study, one group women were examined 42 days postpartum, further improvement in the repeated postpartum ultrasound evaluations after a longer period would be required to confirm the effect of childbirth on PB. In future studies, we will conduct a more scientific and complete study design to have a deeper understanding of PB.

## 5. Conclusion

In conclusion, high-frequency 2D ultrasound with SWE mode, a universally available and convenient examination, is valuable in visualizing the morphology and evaluating dimensions and mechanical properties of PB. The manifestations and measurements of PB are influenced by parity and long-term increased abdominal pressure. Our study preliminarily shows that PB has little effect on levator hiatus area, further researches into the supportive mechanism of the PB on pelvic floor is needed.

## Data availability statement

The original contributions presented in the study are included in the article/supplementary material, further inquiries can be directed to the corresponding authors.

## Ethics statement

The studies involving human participants were reviewed and approved by The Ethics Committee of Shanghai Sixth People’s Hospital Affiliated to Shanghai Jiao Tong University School of Medicine. The patients/participants provided their written informed consent to participate in this study.

## Author contributions

MZ and WS conceived the idea and drafted the manuscript. WB and XW revised the paper. TY worked to organize the topic issue and made contributions to the editorial contents. All authors contributed to the article and approved the submitted version.

## Funding

The study was funded by the National Key Research and Development Program of China (No. 2021YFC2009100, 2021YFC2009101, and 2021YFC2009102) and Shanghai Key Discipline of Medical Imaging Fund (No. 2017 ZZ 02005).

## Conflict of interest

The authors declare that the research was conducted in the absence of any commercial or financial relationships that could be construed as a potential conflict of interest.

## Publisher’s note

All claims expressed in this article are solely those of the authors and do not necessarily represent those of their affiliated organizations, or those of the publisher, the editors and the reviewers. Any product that may be evaluated in this article, or claim that may be made by its manufacturer, is not guaranteed or endorsed by the publisher.
